# CCCTC-Binding Factor Recruitment to the Early Region of the Human Papillomavirus 18 Genome Regulates Viral Oncogene Expression

**DOI:** 10.1128/JVI.00097-15

**Published:** 2015-02-18

**Authors:** Christian Paris, Ieisha Pentland, Ian Groves, David C. Roberts, Simon J. Powis, Nicholas Coleman, Sally Roberts, Joanna L. Parish

**Affiliations:** a University of Birmingham, School of Cancer Sciences, Birmingham, United Kingdom; b University of St. Andrews, School of Medicine, St. Andrews, Fife, United Kingdom; c University of Cambridge, Department of Pathology, Cambridge, United Kingdom

## Abstract

Host cell differentiation-dependent regulation of human papillomavirus (HPV) gene expression is required for productive infection. The host cell CCCTC-binding factor (CTCF) functions in genome-wide chromatin organization and gene regulation. We have identified a conserved CTCF binding site in the E2 open reading frame of high-risk HPV types. Using organotypic raft cultures of primary human keratinocytes containing high-risk HPV18 genomes, we show that CTCF recruitment to this conserved site regulates viral gene expression in differentiating epithelia. Mutation of the CTCF binding site increases the expression of the viral oncoproteins E6 and E7 and promotes host cell proliferation. Loss of CTCF binding results in a reduction of a specific alternatively spliced transcript expressed from the early gene region concomitant with an increase in the abundance of unspliced early transcripts. We conclude that high-risk HPV types have evolved to recruit CTCF to the early gene region to control the balance and complexity of splicing events that regulate viral oncoprotein expression.

**IMPORTANCE** The establishment and maintenance of HPV infection in undifferentiated basal cells of the squamous epithelia requires the activation of a subset of viral genes, termed early genes. The differentiation of infected cells initiates the expression of the late viral transcripts, allowing completion of the virus life cycle. This tightly controlled balance of differentiation-dependent viral gene expression allows the virus to stimulate cellular proliferation to support viral genome replication with minimal activation of the host immune response, promoting virus productivity. Alternative splicing of viral mRNAs further increases the complexity of viral gene expression. In this study, we show that the essential host cell protein CTCF, which functions in genome-wide chromatin organization and gene regulation, is recruited to the HPV genome and plays an essential role in the regulation of early viral gene expression and transcript processing. These data highlight a novel virus-host interaction important for HPV pathogenicity.

## INTRODUCTION

Papillomaviruses are a highly diverse family of small DNA tumor viruses that specifically infect the mucosal and cutaneous epithelium. Human papillomavirus (HPV) types that infect the mucosal epithelium are subdivided into low-risk and high-risk groups, depending on their association with cancer development (1, 2).

Following infection of cells in the basal layer of epithelium, the viral genome is amplified and maintained as a low-copy-number episome (estimated to be between 10 and 200 copies per cell) (3). RNA polymerase II-dependent transcription of the early proteins is initiated from the early promoter located upstream of the E6 open reading frame (ORF) (P_97_ in HPV16 and P_105_ in HPV18 and HPV31) within the viral upstream regulatory region (URR). This drives expression of the E6 and E7 oncoproteins in the basal cells and stimulates continued cellular proliferation. The E7 gene products target members of the retinoblastoma family of proteins, pRb/p105 (4) and p107 (5), which control cell cycle entry in the basal layer. E7 also targets pRb2/p130 (6), which is highly expressed in the upper layers of the epithelium and prevents cell cycle reentry (7). To circumvent increased p53 expression and cell cycle arrest arising from E7 expression, high-risk E6 protein binds p53 and targets it for degradation (8). By promoting cell cycle reentry and delaying differentiation, E6 and E7 facilitate virus replication in cells that normally would have exited the cell cycle. In the upper epithelial layers, the viral genome copy number rises in part as a result of increased production of the viral E1 and E2 proteins (9, 10). Increased E2 expression is thought to repress E6 and E7 production (11), stimulating cellular differentiation and subsequent activation of the differentiation-dependent late promoter (12). This allows the production of transcripts encoding E1^E4, which promotes viral genome amplification (13), and the L1 and L2 capsid proteins (14). This intricate balance and control of early and late gene expression is essential for the completion of the HPV life cycle.

All HPV transcripts are polycistronic. Alternative splicing and polyadenylation of transcripts further regulate HPV early gene expression and increase the repertoire of expressed proteins (14–17). Exactly how splicing of the early transcripts is regulated is not clearly understood, but suboptimal configuration of the 3′ splice sites is thought to allow selection between alternative splice acceptor sites (14). HPV16 also upregulates splicing factors in differentiating epithelium to support late transcript processing (18, 19), highlighting the ability of HPV to manipulate the host environment to control gene expression and coordinate the differentiation-dependent life cycle.

CCCTC-binding factor (CTCF) is a ubiquitous host architectural protein that binds 10,000 to 50,000 sites within the human genome (20). Dynamic, three-dimensional organization of the human genome by CTCF controls numerous genomic processes, including transcription, genetic imprinting, chromatin insulation, and gene splicing (21–25). These functions are coordinated by CTCF through its ability to form long-range interactions, bringing together distant regulatory elements to control gene expression (26), or by forming a roadblock which slows the transcription machinery and alters cotranscriptional RNA splicing (21). Due to the highly complex and regulated nature of HPV gene expression and posttranscriptional processing, we hypothesized that CTCF regulates differentiation-dependent HPV gene expression.

## MATERIALS AND METHODS

### Bioinformatics.

The DNA sequences for each HPV type screened are defined in Table 2. Predictions for CTCF binding sites were made with a combination of CTCF binding site databases (http://insulatordb.uthsc.edu/ and http://bsproteomics.essex.ac.uk:8080/bioinformatics/ctcfbind.htm) or using Storm analysis software. The position weight matrices (PWM) utilized by these analysis tools have been published previously (27–29).

### Plasmids and antibodies.

pUC19-HPV6b, pBR322-HPV11, and pBR322-HPV16 were a gift from E.-M. de Villiers, DKFZ, Germany. pBR322-HPV31 was a gift from L. Laimins, Northwestern University, USA. pGEMII-HPV18 was a gift from F. Stubenrauch, University of Tübingen, Germany, and was used as a template for site-directed mutagenesis (QuikChange II XL; Agilent Technologies, USA) to create pGEMII-HPV18-ΔCTCF that contains three conservative nucleotide substitutions (C^2993^→T, G^3005^→A, T^3020^→C) within the E2 coding region. The plasmid pDrive-SP6-His-CTCF was a gift from D. Farrar (University of Essex, United Kingdom) and encodes human CTCF protein with a 10× histidine tag at the N terminus.

CTCF antibody was purchased from Active Motif (Belgium). FLAG M2 and anti-cytokeratin 1/10 8.60 antibodies were purchased from Sigma-Aldrich (United Kingdom). Anti-cytokeratin 5 D5/16 B4 was purchased from Boehringer Mannheim Biochemica (Switzerland), and loricrin AF62 was from Covance Research Products (United Kingdom). Bromodeoxyuridine (BrdU) and p130 were purchased from Becton Dickinson (United Kingdom). Cyclin B1 H-433, HPV18 E6 (G-7), p53 DO1, and glyceraldehyde-3-phosphate dehydrogenase (GAPDH) were purchased from Santa Cruz Biotechnology (USA). Phospho-histone H3 (P-H3; S10) was purchased from Cell Signaling (USA), and HPV18 E7 (8E2) was from Abcam (United Kingdom). All fluorescent secondary antibodies were purchased from Invitrogen (United Kingdom). Rabbit polyclonal anti-HPV16 E2 antibody was obtained from F. Thierry (Singapore) (9). Monoclonal anti-E1^E4 1D11 (30) and rabbit polyclonal anti-E1^E4 r424 (31) were used to detect HPV18 E1^E4.

### EMSA.

For electrophoretic mobility shift assay (EMSA), DNA fragments were amplified with a forward primer containing an M13-overhang (sequences are available upon request) using master mix S (PeqLab, Germany). The products of the first PCR then were amplified in a second PCR using a 6-carboxyfluorescein (FAM)-labeled M13 forward primer. CTCF protein was produced in an *in vitro* transcription translation reaction using the TNT SP6 high-yield wheat germ protein expression system (Promega, United Kingdom).

Two μl of FAM-labeled DNA was incubated with 1 μl CTCF protein in a 10-μl reaction mixture containing 0.5% NP-40, 50 mM KCl, 10 mM Tris-HCl, pH 7.5, 0.1 μg/μl poly(dI-dC), 5% Ficoll 400, 1 mM phenylmethylsulfonyl fluoride (PMSF), and 0.1 mM dithiothreitol (DTT). Samples were incubated at room temperature for 1 h before separation on a 4.5% native polyacrylamide gel. FAM fluorescence was imaged at 520 nm using a Typhoon FLA7000 (GE Healthcare Life Sciences, United Kingdom).

### ChIP.

Chromatin immunoprecipitation (ChIP) assays were carried out using the ChIP-IT express enzymatic ChIP kit (Active Motif) by following the manufacturer's instructions. Cells were fixed in 1% formaldehyde for 3 min at room temperature, and nuclei were released by 40 strokes in a tight dounce homogenizer. DNA was purified using a GenElute PCR cleanup kit (Sigma-Aldrich). ChIP efficiency was assessed by quantitative PCR (qPCR) using SensiMix SYBR master mix (Bioline, London, United Kingdom) using an MXPro 3000 (Agilent Technologies). Primer sequences used are available upon request. Cycle threshold (*C_T_*) values were calculated at a constant threshold for each experiment, and the percentage of input DNA was calculated using the standard curve.

### Keratinocyte culture, transfection, and organotypic raft culture.

W12 cells containing episomal HPV16 genomes were cultured as previously described (32). The transfection of normal primary foreskin keratinocytes (HFKs) from neonatal foreskin epithelia (ethical approval number 06/Q1702/45) was performed in S. Roberts' laboratory by J. Parish as previously described (31, 33). To eliminate donor-specific effects, 2 donor lines were used: one produced as described above and one commercially available HFK line (Clonetics, Lonza Group Ltd., Basel, Switzerland). Emerging cell colonies were pooled and expanded as previously described (34). Genomes were extracted from each line and sequenced to ensure that the mutations were present in the mutant genome-containing lines. Organotypic rafts were prepared (31) and cultured for 14 days in E medium without epidermal growth factor to allow cellular stratification. Sixteen hours prior to harvesting, 20 μM BrdU was added to the growth medium. Rafts then were fixed in 3.7% formaldehyde (Sigma-Aldrich) and paraffin embedded prior to sectioning (Propath Ltd., Hereford, United Kingdom).

### Cell growth assay.

A total of 1 × 10^5^ terminally gamma-irradiated J2-3T3 fibroblasts were seeded to each well of three 12-well tissue culture microtiter plates and left to adhere. Wells then were seeded with 1 × 10^4^ HFK lines in triplicate. The growth of cells was measured at days 1, 3, and 5 following removal of J2-3T3 fibroblasts by washing with EDTA and phosphate-buffered saline (PBS). Five hundred μl growth medium and 50 μl CCK-8 reagent (Dojindo Molecular Technologies, Inc.) were added to each well, and the plate was incubated at 37°C for 2 to 4 h. Absorbance was read at 450 nm using an iMark microplate reader (Bio-Rad). Wells that contained J2 3T3 fibroblasts but not HFK were used as a blank for each plate.

### Immunofluorescence.

Four-μm sections of organotypic cultures were placed on polylysine-coated slides and incubated at 50°C for 30 min. One section from each raft was stained with hematoxylin and eosin for morphological analysis. Antigens were retrieved using an agitated low-temperature method, as previously described (35), following immersion in Histoclear (National Diagnostics, Yorkshire, United Kingdom). Slides were blocked with 20% heat-inactivated goat serum and 0.1% bovine serum albumin (BSA) in PBS for 1 h at room temperature. Primary antibodies were incubated on the slides overnight at 4°C. Incubation in secondary antibody subsequently was performed at 37°C for 1 h. DNA was stained with Hoechst 33342 before mounting in Fluoroshield (Sigma-Aldrich). Microscopic analysis was performed in a Nikon E600 epifluorescence microscope, and images were captured using a Nikon DXM1200F digital camera.

### Chromogenic *in situ* hybridization (C-ISH).

Nuclei positive for HPV DNA amplification in raft sections were detected with a biotin-conjugated high-risk HPV DNA-specific probe using Leica Bond-Max technology, as described by the manufacturer (Leica Microsystems, Milton Keynes, United Kingdom).

### Episome copy number determination.

Southern blotting was performed as described previously (36). For qPCR analysis, relative quantities of HPV18 genome in total DNA, amplified with primers 5′-TTATAGGCGAGCCCAAAAAC-3′ and 5′-CCAATCTCCCCCTTCATCTAT-3′, were normalized against the TLR2 locus at chromosome 4q32 using the Pfaffl comparative *C_T_* method (37).

### Transcript analysis.

RNA was extracted from 14-day-old HFK raft cultures using RNA-STAT 60 (AMS Biotechnology Ltd., United Kingdom). Five μg of RNA was treated with 1 U of RQ1 DNase (Promega) for 30 min at 37°C, which subsequently was inactivated for 10 min. Reverse transcription was performed using a Tetro cDNA synthesis kit (Bioline). Two μl of cDNA was used for the amplification of HPV transcripts using the primers listed in Table 1. Products were separated by electrophoresis, and the relative intensity of each product was measured using ImageJ.

**TABLE 1:** Primers used for HPV18 transcript analysis

Amplicon	Primer (5′ to 3′)		Reference or source
	Forward	Reverse	
121-295	ATCCAACACGGCGACCCTAC	GCAGCATGCGGTATACTGTCTCTA	14 and this study
121-3517	ATCCAACACGGCGACCCTAC	ACGGACACGGTGCTGGAA	14
E1F1/E4R	CAACAATGGCTGATCCAGAAG	AGGTCCACAATGCTGCTTCT	15

### Statistical analysis.

A two-tailed, unpaired student's *t* test was used to determine statistical significance.

## RESULTS

### Identification of CTCF binding sites in alpha-HPV genomes by bioinformatic analysis.

CTCF binding sites in the genomes of low-risk HPV types 6b and 11 and high-risk HPV types 16, 18, and 31 were predicted using open access databases and Storm analysis software (Table 2). These motif identification tools use a combination PWM previously described (27–29). As hypothesized, all of the HPV types tested were predicted to bind CTCF at multiple sites, although the number of predicted binding sites within different HPV types varied, ranging from six sites in HPV16 to 11 sites in HPV6b and HPV18. Numerous predicted binding sites clustered within the late gene region of all types studied. An additional site was identified in the E2 open reading frame (ORF) that was conserved in the high-risk but not in the low-risk viral types.

**TABLE 2:** Prediction of CTCF binding in various HPV types and relative *in vitro* binding affinity

Class, type,^*a*^ and predicted motif	Fragment tested	Name	Motif sequence	Confirmed *in vitro*	Relative strength of binding
High risk					
HPV18 (AY262282.1)					
843	754-943	18_1	ATTCCAGCAGCTGTTTCTGA	No	ND
1205	1102-1297	18_2	CCATTAGGGG	No	ND
2989	2926-3117	18_3	AAACCACCAGGTGGTGCCAG	Yes	Strong^*b*^
3487	3381-3575	18_4	CGGTGAGGGG	No	ND
3620	3527-3718	18_5	TTGCCTGTAGGTGTAGCTGC	Yes	Medium
4505 and 4537	4440-4638	18_6 and 18_7	CGTCCCCCAGTGGT/GTAACAATAGATGGGTCTGT	Yes	Two medium bands
None	4947-5155	18_8	NA	No	ND
None	5045-5253	18_9	NA	No	ND
5473	5381-5577	18_10	CATACAGAGG	Yes	Medium
5767	5655-5850	18_11	CACCACCTGCAGGA	Yes	Medium
HPV16 (NC_001526.2)					
1282	1216-1405	16_1	AACTCAGCAGATGTTACAGG	No	ND
2915	2852-3049	16_2	TAACCACCAAGTGGTGCCAA	Yes	Strong^*b*^
5118	5000-5207	16_3	CGCCTAGAGG	Yes	Weak
6127	6051-6278	16_4	CCTATAGGGG	Yes	Weak
6514	6426-6600	16_5	GAACCACTAGGTGTAGGAA	Yes	Weak
6859	6772-6957	16_6	CTCCCCCAGGAGGC	Yes	Weak
HPV31 (J04353.1)					
615	534-713	31_1	ATAACAGTGGAGGTCAGTT	No	ND
885	804-1008	31_2	TGGGGAGGGG	No	ND
1093	1029-1200	31_3	CATGCAGAGG	No	ND
1277	1182-1374	31_4	AACGCAGCAGATGGTACAGG	No	ND
2332	2230-2406	31_5	CAACCACTGGCTGATGCTAA	No	ND
2412	2357-2531	31_6	AATGCACTAGATGGCAACC	Yes	Strong^*b*^
2853	2801-3015	31_7	TAACCACCAGGTGGTGCCAG	Yes	Strong^*b*^
None	2894-3093	31_8	NA	No	ND
5179	5077-5273	31_9	CCTTTAGGGG	Yes	Strong
6431	6354-6540	31_10	CTACACCTAGCGGC	Yes	Medium
Low risk					
HPV6b (NC_001355.1)					
1357	1251-1460	6b_1	CATACAGAGG	No	ND
None	2801-3007	6b_2	NA	No	ND
None	2887-3101	6b_3	NA	No	ND
4789	4715-4913	6b_4	TGTGCAGGGG	No	ND
5018, 4987	4913-5102	6b_5 and 6b_6	CTATCACTAGATGATACCA/CCTATAGAGG	No	ND
5424	5317-5515	6b_7	GCAGCCACAAGAGGGTGCAT	Yes	Strong
6109	5995-6199	6b_8	CAGCCATTAGGTGTGGGTGT	No	ND
6263	6179-6382	6b_9	CCCAAAGGGG	Yes	Medium
7205, 7256	7155-7380	6b_10 and 6b_11	CGAATAGAGG/CGTTTAGGGG	No	ND
HPV11 (FR872717.1)					
1357	1295-1494	11_1	CATAGAGAGG	No	ND
None	2801-3003	11_2	NA	No	ND
None	2900-3104	11_3	NA	No	ND
4058	3930-4153	11_4	TGCAAAGGGG	Yes	Medium
4781	4709-4898	11_5	TGTGTAGGGG	No	ND
4920	4844-5041	11_6	CCACCTGTGGAGGCCAGTG	Yes	Weak
5415	5330-5501	11_7	GCAGCCACTAGAGGGTGCAG	Yes	Strong
6310	6243-6428	11_8	GTTCCAACGGGGGGCAGTC	Yes	Weak
6635	6544-6738	11_9	GAGCCACTAGGTGTATGTA	Yes	Weak, smear
6979	6872-7074	11_10	CCTCCACCAAATGGTACACT	No	ND

a The accession number of each HPV genome analyzed is indicated. The position in the viral genome of the first nucleotide of each predicted motif is given along with the specific fragment tested by EMSA. Where a fragment was tested that did not contain a predicted motif, no position is given (none). Each fragment tested is named by HPV type followed by the order of position in the genome starting at position 1 in the URR. The sequence of each motif is given (NA indicates that a motif was not predicted). Fragments were tested for binding *in vitro* by EMSA. The relative strength of binding to each fragment was assessed qualitatively by comparison to the proportion of c-Myc-positive control DNA bound by CTCF in the same assay (weak, <50% binding; medium, 50 to 75% binding; strong, >75% binding; ND, none detected). All EMSA experiments were repeated at least three times, and the strength of binding reflects the relative binding strength achieved in all repeats.

b CTCF binding site within the E2 ORF that is conserved in all high-risk HPV types tested.

### Verification of CTCF binding sites.

**FIG 1:**
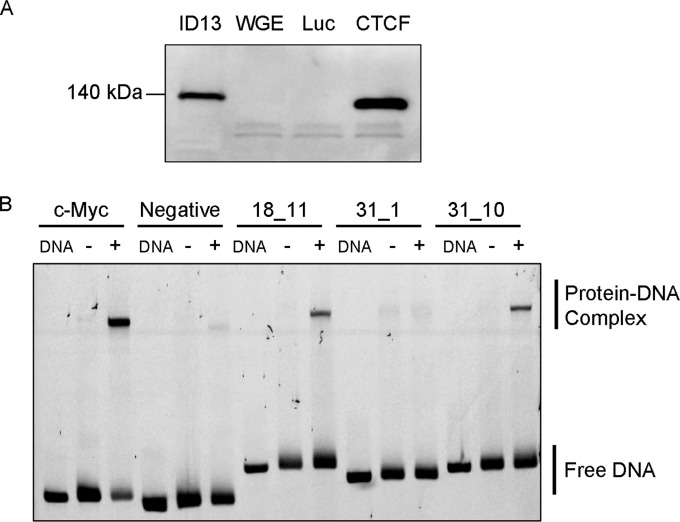
*In vitro* analysis of the association of CTCF with HPV genomes. (A) Western blot analysis of *in vitro*-translated CTCF protein. Lysate from ID13 (mouse) cells known to express CTCF was loaded as a positive control alongside wheat germ extract (WGE) and WGE used to translate luciferase (Luc) or CTCF protein. A band running at approximately 140 kDa was present in the ID13 cell lysate, and a slightly smaller band was present in the *in vitro*-translated CTCF reaction. Human CTCF is an 82-kDa protein but runs at approximately 130 kDa on SDS-PAGE (71), whereas the mouse homologue is slightly larger. (B) An example of an EMSA of CTCF binding to predicted BPV DNA fragments. DNA fragments were amplified and labeled with FAM by PCR. Fragments were mixed with binding buffer only (DNA), *in vitro*-translated luciferase protein (Luc) (−), or *in vitro*-translated CTCF protein (+), and protein-DNA complexes were separated on a native acrylamide gel. Free DNA is indicated at the bottom of the gel and protein-DNA complexes near the top. Each fragment was tested a minimum of three times, and the combined results are shown in Table 2. Fragments from the c-Myc locus (positive control), a region of the BPV-1 genome that is known not to bind CTCF (negative control), and fragment 11 from HPV18 and fragments 1 and 10 from HPV31 are shown in the representative EMSA. 18_11 and 31_10 bound CTCF with medium strength (50 to 75% binding compared to the c-Myc positive control), and 31_1 did not bind CTCF *in vitro*.

**FIG 2:**
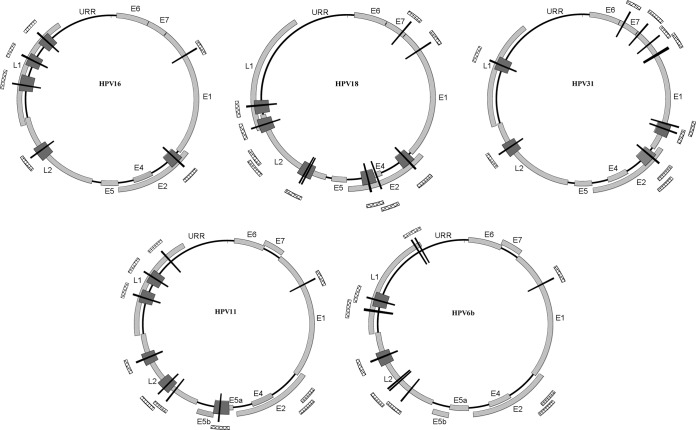
Summary of *in silico*-predicted CTCF binding sites and *in vitro* analysis. Graphical representations of the HPV16, HPV18, HPV31, HPV11, and HPV6b genomes are shown. ORFs are indicated on each genome (light gray). Predicted CTCF binding sites are represented by the black bars. The hashed bars on the periphery of each genome highlight fragments tested by EMSA, and the dark gray bars on each genome indicate those fragments that bound CTCF *in vitro*.

To confirm our *in silico* analysis, CTCF binding was assessed *in vitro* by EMSA. Approximately 200-bp DNA fragments containing the predicted binding motifs were incubated with CTCF protein (Fig. 1A), and complexes were separated by electrophoresis (Fig. 1B). A region of the c-Myc promoter, previously shown to bind CTCF (38), and a fragment of the BPV1 genome not predicted to bind CTCF were included as controls. Fragments also were incubated with wheat germ extract alone and *in vitro*-translated luciferase to control for nonspecific binding of proteins. Fragments were tested a minimum of three times, and the relative strength of binding compared to that of the c-Myc positive-control DNA fragment was estimated (Table 2 and Fig. 2).

The binding maps presented in Fig. 2 show conservation of CTCF binding between HPV types. All types contain a cluster of CTCF binding sites within the late gene region, ranging from 2 binding sites in HPV6b to 4 binding sites in HPV16. Furthermore, the conservation of one to two CTCF binding sites within (or close to) the E2 ORF of the high-risk HPV types was confirmed. Binding in this region was not detected in HPV6b or HPV11 with fragments amplified from this region (Table 2). The conservation of CTCF binding sites between HPV types supports our hypothesis that CTCF recruitment is an important virus-host interaction in the HPV life cycle.

### CTCF associates with HPV16 and HPV18 genomes.

**FIG 3:**
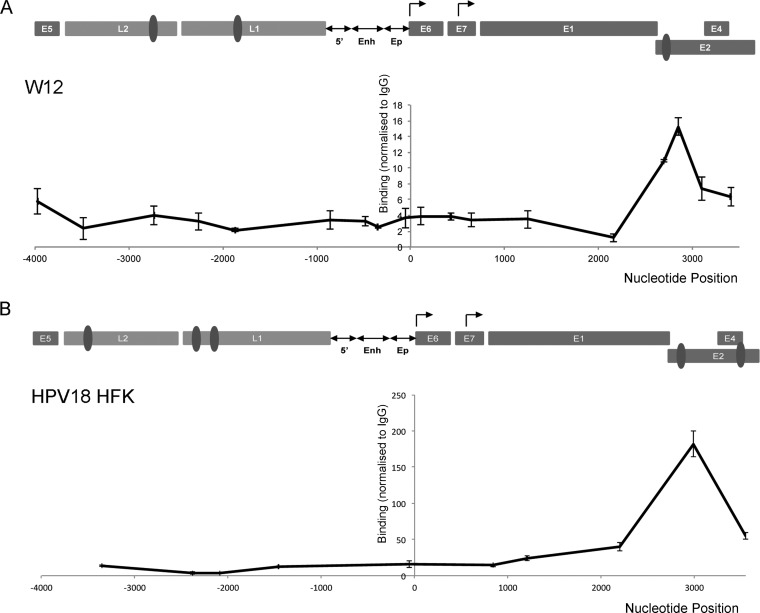
Association of CTCF with HPV genomes. Chromatin extracted from HPV16-positive W12 cells (A) and HPV18-positive HFKs (B) was immunoprecipitated with control antibody (rabbit IgG for W12 and FLAG M2 antibody for HPV18 HFKs) or CTCF-specific antibody. Coprecipitating DNA was analyzed by qPCR. The *x* axes indicate the position in the HPV genome amplified, and each data point represents the central point in each amplicon. A graphical representation of the HPV genome is shown above each data set, which has been linearized for ease of presentation (Enh, enhancer; Ep, early promoter). The CTCF binding sites verified by EMSA (Fig. 1 and Table 2) are indicated (dark gray ovals). Binding efficiency was normalized to negative-control antibody using the ΔΔ*C_T_* method. The data represent the means and standard errors from three independent repeats.

We next used HPV16 and HPV18 genome-containing cells to ascertain whether CTCF associates with the viral genome in cells. W12 cells, derived from a low-grade cervical squamous epithelial lesion, contain ∼100 episomal HPV16 genome copies/cell (39, 40), and HPV18-transfected HFKs contain ∼200 episomal HPV18 copies/cell (see Fig. 5B). CTCF association with the HPV genomes was determined by ChIP followed by qPCR. In both HPV16 and HPV18 genome-containing cells grown in monolayer, we noted a significant enrichment of CTCF binding within the E2 ORF, coinciding with the CTCF binding site conserved in high-risk HPV types but not in low-risk types (Fig. 3). In contrast, we failed to detect CTCF binding to the late gene region in either HPV16 or HPV18 genome-containing model systems.

### Loss of CTCF binding to the HPV18 genome does not alter episome establishment or proliferation of primary human foreskin keratinocytes.

**FIG 4:**
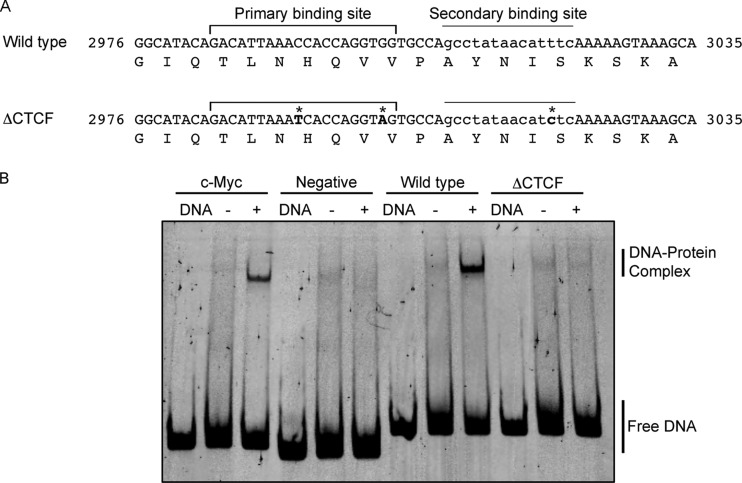
Mutation of the CTCF binding site at position 2989 in HPV18. (A) Wild-type HPV18 sequence between nucleotides 2976 and 3035 showing the primary CTCF binding site starting at nucleotide 2989 and the secondary binding site in lowercase. The amino acid sequence of E2 protein in this region is shown below the DNA sequence. The 3 conservative nucleotide substitutions created in the mutated ΔCTCF HPV18 genome (C→T^2993^, G→A^3002^, and T→C^3020^) are indicated (*). (B) Abrogation of CTCF binding was assessed by EMSA. The CTCF binding region of the c-Myc locus (positive control), a region of the BPV-1 genome that does not contain CTCF binding sites (negative control), and the CTCF binding regions in the E2 ORF in wild-type and ΔCTCF mutant genomes were amplified and FAM labeled by PCR. DNA fragments were mixed with binding buffer (DNA) alone or with *in vitro*-translated luciferase (−) or CTCF (+), and complexes were separated on a native acrylamide gel. In agreement with data presented in Table 2, CTCF bound strongly to the wild-type HPV18 (18_3) fragment compared to the positive control; however, binding of CTCF to the ΔCTCF mutant fragment was severely disrupted.

**FIG 5:**
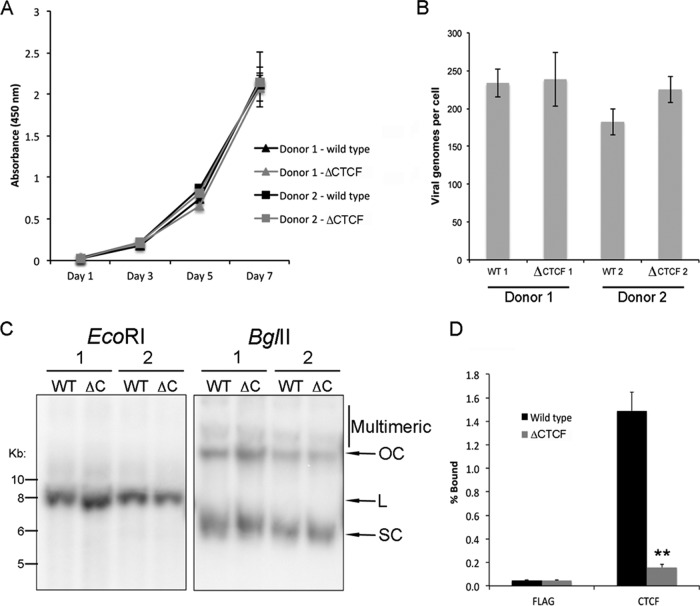
Creation of HPV18 wild-type and ΔCTCF mutant human foreskin keratinocyte lines. HFKs established from two independent donors were transfected with WT or ΔCTCF HPV18 genomes. (A) Analysis of growth kinetics using a CCK-8 metabolic assay. Cells were seeded at equal density at day 0, and the growth of each line was measured at days 1, 3, 5, and 7. The data show the means and standard errors from two independent experiments performed in triplicate. (B) HPV18 genome copy number was determined by qPCR analysis of DpnI-digested DNA extracted from each line using the Pfaffl comparative *C_T_* method and normalized against the *TLR2* locus (37). Data show the means and standard errors from three independent repeats (donor 1, *P* = 0.9; donor 2, *P* = 0.2). (C) HPV18 genome status was determined by Southern blotting from extracted DNA from donor 1 and donor 2 transfected with either wild-type (WT) or ΔCTCF mutant (ΔC) HPV18 genomes (OC, open circle; L, linear; SC, supercoiled). DNA was linearized with EcoRI, producing a single band of similar intensity running at approximately 8 kbp, demonstrating the maintenance of viral episomes at a similar copy number in each line. Digestion with BglII shows minimal multimeric/integrated HPV genomes in all lines. (D) Abrogation of CTCF binding by mutation of the CTCF binding site was determined by ChIP. Chromatin was either immunoprecipitated with FLAG (negative control) or CTCF antibody, and the percentage of bound HPV18 genome was determined by qPCR with primers that flank the CTCF binding site at position 2989. A significant decrease in CTCF binding was observed in ΔCTCF HPV18 compared to that of the wild type (**, *P* = 0.01). The data shown represent the means and standard errors from two independent repeats performed in duplicate (donor 1; donor 2 showed a similar decrease in CTCF binding).

To assess the biological function of CTCF binding within the E2 ORF, mutations were introduced into the HPV18 genome to prevent CTCF binding (Fig. 4A). Three nucleotide substitutions were introduced into the predicted binding site that did not alter the amino acid coding sequence of E2 (ΔCTCF HPV18). It should be noted that CTCF also has the potential to bind to the cDNA strand within this region (at the sequence 5′ CACCACCTGGTGGT 3′), although the mutations introduced also would affect binding at this site. We observed a near-complete loss of CTCF binding to the ΔCTCF HPV18 sequence in EMSA, confirming that the mutations prevented CTCF binding (Fig. 4B). HFKs were transfected with recircularized wild-type (WT) or ΔCTCF HPV18 genomes, and immortalized lines were established. To account for donor-specific effects, cells from two independent donors were transfected, and all downstream analyses were performed on both lines. No significant differences in cellular morphology (data not shown) or growth were observed between WT and ΔCTCF lines (Fig. 5A). The physical state of the HPV genomes was determined by Southern blotting and qPCR. Both WT and ΔCTCF HPV18 lines were shown to contain episomal HPV genomes at a similar copy number of approximately 200 copies/cell (Fig. 5B and C). Importantly, we demonstrated a 10-fold reduction in CTCF binding to ΔCTCF HPV18 genomes compared to the level for the WT (Fig. 5D).

### Loss of CTCF binding induces a hyperproliferative phenotype in organotypic culture.

**FIG 6:**
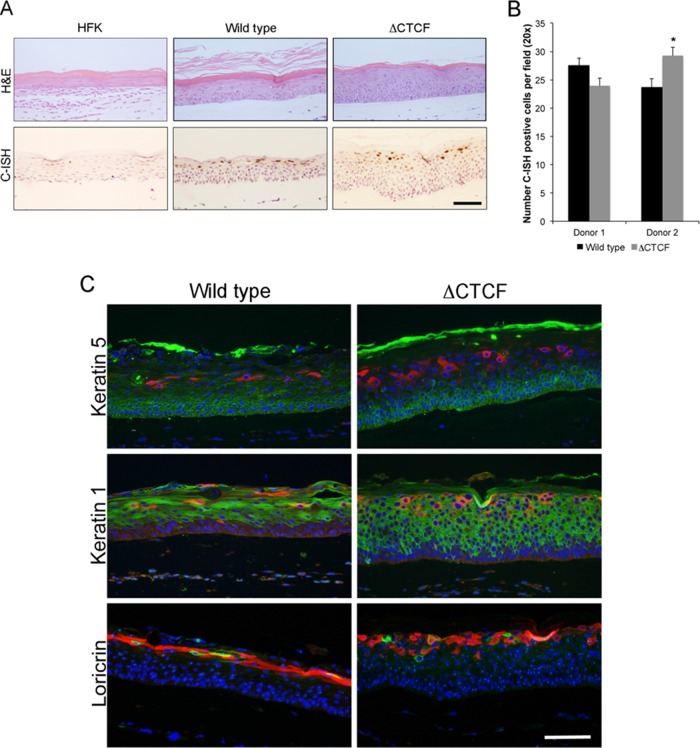
Morphology and differentiation of HPV18 ΔCTCF organotypic raft cultures. (A, upper) Organotypic raft cultures of HFK, WT HPV18, and ΔCTCF HPV18 lines were fixed at day 14, and sections were stained with hematoxylin and eosin to assess morphology (upper). (Lower) Sections were stained by C-ISH to qualitatively assess viral genome amplification. Brown nuclear staining is present in cells with amplified HPV18 genomes. Scale bar, 10 μm. (B) The number of cells positive for C-ISH in wild-type and ΔCTCF HPV18 sections was counted in 10 fields of vision from sections of three independent raft cultures from each line (*n* = 30). The data are shown as means and standard errors. (C) Sections were stained with antibodies specific for keratin 5 (green; upper), keratin 1 (green; middle), or loricrin (red; lower). Sections were counterstained with Hoechst to highlight the nuclei (blue) and E1^E4 antibody to highlight productive areas of each section (red in upper and middle panels [rabbit antibody r424], green in the lower panel [mouse antibody 1D11]). Scale bar, 10 μm.

To assess the biological function of CTCF recruitment to the HPV18 genome in differentiating epithelium, WT and ΔCTCF HPV18 HFK lines were grown in organotypic raft culture. Formaldehyde-fixed rafts were paraffin embedded and sectioned. Sections were stained with hematoxylin and eosin to assess morphology (Fig. 6A). As previously described, the WT HPV18 genome-containing rafts were increased in thickness, and mitotic cells were visible in the lower and upper suprabasal layers of the rafts compared to rafts derived from HFKs that did not contain HPV18 genomes (13). This phenotype was enhanced in ΔCTCF HPV18 rafts, which were consistently thicker, indicating increased cellular proliferation. Alongside these experiments, viral genome amplification was assessed by chromogenic *in situ* hybridization (C-ISH). No consistent differences were observed in the number of cells with amplified HPV genomes between WT and ΔCTCF HPV18 rafts, demonstrating that CTCF recruitment has a minimal role in viral genome amplification (Fig. 6A and B).

### Increased S phase and G_2_ entry is caused by loss of CTCF binding.

The increase in hyperproliferation in ΔCTCF HPV18 rafts could be explained either by delayed epithelial differentiation or by increased S phase entry. To assess molecular differentiation, raft sections were stained for markers of undifferentiated keratinocytes (keratin 5), early differentiation (keratin 1), and late differentiation (loricrin) alongside E1^E4, a marker of the productive phase of the HPV life cycle (Fig. 6C). Expression patterns of keratin 5, keratin 1, and loricrin were similar between WT and ΔCTCF HPV18 organotypic cultures, with keratin 5 confined to the basal and parabasal layers with some nonspecific staining visible in the cornified layer of the epithelium; keratin 1 and loricrin were expressed in the suprabasal and upper layers, respectively. However, keratin 1 and loricrin staining highlighted differences in the morphology of cells in the suprabasal and upper layers of the epithelium; rather than a flattening of these cells in the upper layers, as can be seen in the WT HPV18 sections, the cells appeared to maintain a rounded morphology. This difference in morphology also is visible in the hematoxylin- and eosin-stained sections shown in Fig. 6A.

**FIG 7:**
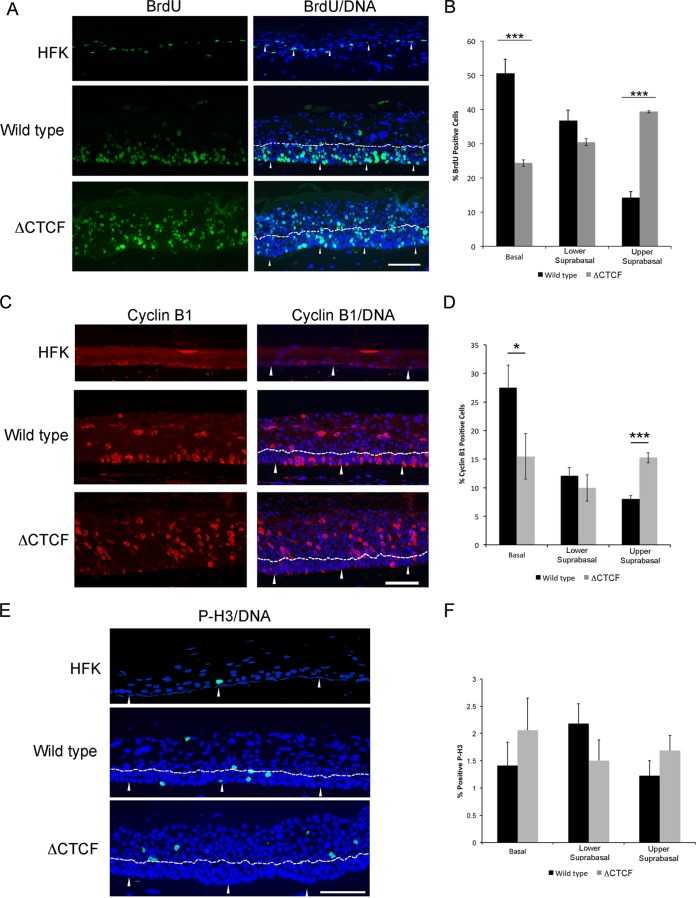
Cell cycle entry in wild-type and ΔCTCF mutant HPV18 genome-containing organotypic raft sections. Sections were stained with anti-BrdU (green) (A), cyclin B1 (red) (C), and phospho-histone H3 (green) (E). DNA was stained with Hoechst to highlight the nuclei (blue). Representative sections of WT and ΔCTCF HPV18 genome-containing HFK rafts are shown. The white arrows indicate the basal layer, and the lower suprabasal/upper suprabasal boundary is highlighted with a dashed line. Scale bar, 10 μm. The percentage of cells stained positive for nuclear BrdU (B), cytoplasmic cyclin B1 (D), and phospho-histone H3 (F) in the basal, lower suprabasal (parabasal and lower spinous), and upper suprabasal (upper spinous and granular) layers of 15 fields of view of 3 independent rafts (*n* = 45) from each donor was determined. The data represent the means and standard errors. (B) A significant reduction in BrdU incorporation is observed in the basal layer of ΔCTCF HPV18 lines (***, *P* = 0.002), a small reduction is observed in the lower suprabasal compartment that did not reach significance (*P* = 0.07), and a significant increase in BrdU incorporation is observed in the upper suprabasal layers of the ΔCTCF HPV18 rafts compared to the wild type (***, *P* = 0.0002). (D) A significant reduction in cyclin B1-positive cells is observed in the basal layer of ΔCTCF HPV18 lines (*, *P* = 0.04), no difference is observed in the suprabasal compartment, and a significant increase in cyclin B1-positive cells is observed in the upper layers of the ΔCTCF HPV18 rafts compared to those of the wild type (***, *P* = 0.00006). (F) No significant differences in P-H3-positive cells were observed.

BrdU incorporation was used to assess cell cycle entry and cellular DNA replication. BrdU-positive cells were confined to the basal layer in rafts derived from untransfected donor keratinocytes (Fig. 7A). Increased S phase entry was observed in the basal and suprabasal layers of the WT HPV18 rafts, as previously reported (36). In contrast, ΔCTCF HPV18 rafts displayed a significant decrease in S phase entry in the basal layer compared to that of the WT. A decrease in the percentage of BrdU-positive cells was observed in the lower suprabasal layers of ΔCTCF HPV18 rafts compared to that of the WT, although this did not reach significance. In contrast, a significant increase in the percentage of BrdU-positive cells was observed in the upper suprabasal layers of the ΔCTCF HPV18 rafts (Fig. 7B).

Raft sections also were stained for cyclin B1 and P-H3 (Ser10) as markers of G_2_ and mitotic entry, respectively (Fig. 7C and E). In agreement with the BrdU incorporation analysis (Fig. 7A and B), a decrease in cells positive for cytoplasmic cyclin B1 was observed in the basal layer of ΔCTCF HPV18 lines compared to that of the WT. No significant difference was observed in the lower suprabasal compartment, but an increase in cytoplasmic cyclin B1 in the upper suprabasal layers was noted (Fig. 7C and D). In contrast, there was no difference in the number of cells positive for P-H3 in WT and ΔCTCF HPV18 structures (Fig. 7E and F). Taken together, these data indicate that there is an increase in cell cycle entry with a corresponding increase in S and G_2_ phases in the upper layers of the epithelium of ΔCTCF HPV18 cells. The cells appear to arrest at G_2_ phase as an increase in mitotic entry is not observed. These data provide evidence that loss of CTCF binding within the HPV18 E2 ORF leads to a delay in cell cycle exit and an enhanced hyperproliferative phenotype.

### CTCF binding within the E2 ORF controls the expression of viral oncoproteins E6 and E7.

**FIG 8:**
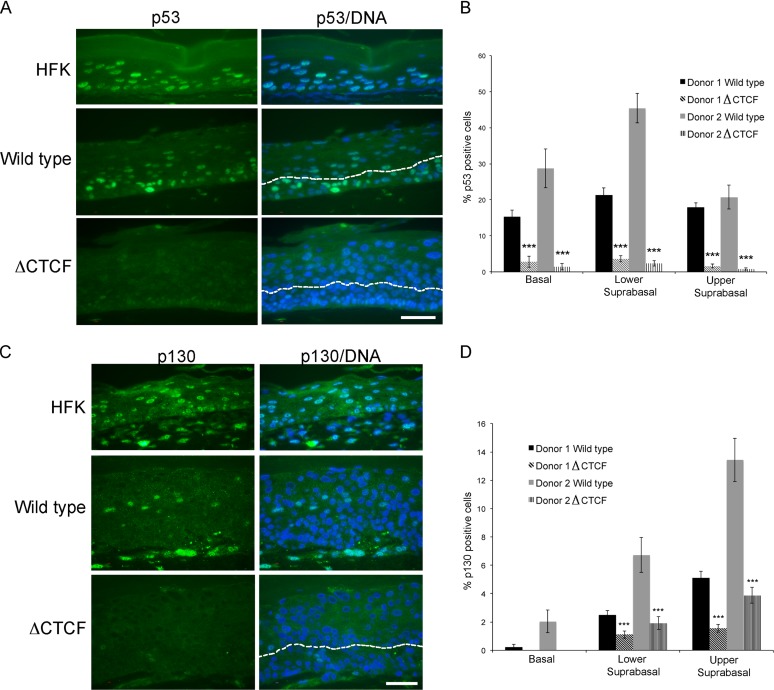
Analysis of p53 and p130 degradation in wild-type and ΔCTCF HPV18 organotypic raft sections. (A) Sections were stained with p53-specific antibody (green), and DNA was stained with Hoechst (blue). The white arrows indicate the basal layer, and the lower suprabasal/upper suprabasal boundary is highlighted with a dashed line. Scale bar, 5 μm. (B) The percentage of cells positive for nuclear p53 staining in the basal, lower suprabasal (parabasal and lower spinous), and upper suprabasal (upper spinous and granular) layers of 15 fields of view of 3 independent rafts (*n* = 45) from each donor was determined. The data represent the means and standard errors. A significant reduction in p53-positive cells is observed in all layers of rafts derived from the ΔCTCF HPV18 lines (***, *P* < 0.0005). (C) Sections were stained with p130-specific antibody (green), and DNA was stained with Hoechst (blue). The white arrows indicate the basal layer, and the lower suprabasal/upper suprabasal boundary is highlighted with a dashed line. Scale bar, 5 μm. (D) The percentage of cells positive for nuclear p130 staining in the basal, lower suprabasal (parabasal and lower spinous), and upper suprabasal (upper spinous and granular) layers of 15 fields of view of 3 independent rafts (*n* = 45) from each donor was determined. The data represent the means and standard errors. A significant reduction in p130-positive cells is observed in all layers of rafts derived from the ΔCTCF HPV18 lines (***, *P* < 0.001).

The increased cell cycle entry and hyperproliferation observed in the organotypic raft cultures derived from HFK lines maintaining ΔCTCF HPV18 genomes could be due to an increase in the expression of E6 and E7 viral oncoproteins. Detection of these proteins by immunostaining currently is not possible; therefore, raft sections were stained with surrogate markers, p53 as a marker for E6 expression and pRb family member p130 for E7 expression (8, 41). Cells stained positive for p53 in WT HPV18 raft sections were apparent throughout the epithelia as previously reported (42), albeit at a noticeably decreased level compared to that of rafts derived from untransfected HFKs (Fig. 8A). In contrast, p53-positive cells were undetectable in rafts derived from ΔCTCF HPV18 lines (Fig. 8A and B). This observation is consistent with an increase in E6 protein levels in ΔCTCF HPV18 compared to that of the WT, resulting in a decrease in detectable p53 protein. Similarly, immunostaining with p130-specific antibodies revealed significant differences between WT and ΔCTCF HPV18 rafts (Fig. 8C and D). In wild-type HPV18 rafts, p130-positive cells were confined to the upper layers, as previously shown (42) and in contrast to HPV-negative HFK raft sections, where cells stained positive for p130 in the parabasal and lower and upper suprabasal layers. However, immunostaining of p130 in the ΔCTCF HPV18 raft sections revealed an almost complete loss of p130-positive cells in the upper layers, suggesting increased and prolonged expression of E7 protein in the ΔCTCF HPV18 rafts compared to that of the WT.

**FIG 9:**
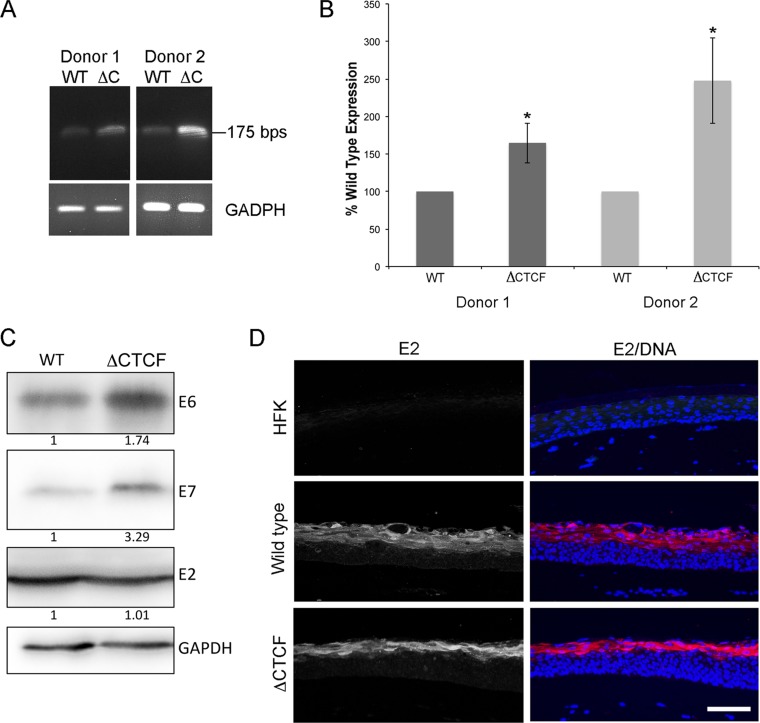
Analysis of unspliced E6E7 transcript and protein expression in organotypic raft culture. RNA extracted from 14-day-old raft cultures was converted to cDNA and amplified between nucleotides 121 and 295. The products of this PCR are unspliced early transcripts (14). Amplification of GADPH from the same samples is shown as a loading control. Products were separated by electrophoresis (A) and quantified by densitometry using ImageJ (B). An increase in E6E7 transcript was observed in ΔCTCF HPV18 lines established from individual donors (*, *P* = 0.03 for donor 1 and donor 2). (C) Proteins extracted from raft cultures were analyzed by Western blotting. Fold increase in virus protein expression compared to the wild type (normalized to GAPDH protein) is indicated below each membrane section. The images shown are representative of three technical repeats of lysates extracted from two independent donor lines. (D) E2 protein localization (red in the merged image; DNA is blue) in raft sections of HFK, wild-type, and ΔCTCF HPV18 genome-containing lines. The images shown are representative of two independent raft cultures of each individual donor line. Scale bar, 10 μm.

Since p53 and p130 expression only provide an indication of E6 and E7 activity, we also quantified expression of early transcripts that have the potential to encode E6 and E7 by reverse transcriptase PCR (RT-PCR). As expected, the relative abundance of unspliced E6E7 transcripts in ΔCTCF HPV18 raft cultures was significantly increased compared to that of the WT (Fig. 9A and B). E6E7 transcript levels also were measured by qPCR using the same primer set as that described above and compared to the human RPLPO gene (Life Technologies). A ratio of E6E7 transcript to RPLPO transcript in HPV18 wild-type and ΔCTCF rafts was calculated using the Livak 2^ΔΔ*CT*^ method. Donor 1 was shown to have a 21.19-fold increase (±10.48-fold standard errors [SE]) and donor 2 had a 44.08-fold increase (±26.95-fold SE) in E6E7 transcript in the HPV18 ΔCTCF rafts compared to wild-type levels. In addition, Western blot analysis of protein extracts from raft cultures harvested at day 14 demonstrated a clear increase in E6 and E7 protein levels (Fig. 9C), which was consistent in both donor lines.

The increase in E6E7 unspliced transcript could be due to an increase in the activity of the early promoter. HPV-encoded E2 protein is known to repress the activity of this promoter, and changes in E2 expression could affect early promoter activity (43–45). To determine whether the expression level of E2 protein was affected by the mutations introduced into the E2 ORF in the ΔCTCF HPV18 genome, E2 protein levels in raft lysates were detected by Western blotting, and no changes were observed (Fig. 9C). Furthermore, immunostaining of sections showed E2 staining in the intermediate and upper layers of the WT raft cultures, with obvious cytoplasmic and nuclear localization. As previously described, E2 staining was not detected in the basal and lower suprabasal cells, presumably because E2 protein levels are below the level of detection (9). No staining was detected in the HPV-negative HFK raft control, demonstrating specificity of the antibody. An equal intensity of E2-specific signal was observed in the upper layers of wild-type and ΔCTCF HPV18 rafts, although a delay in E2 expression was consistently observed in ΔCTCF HPV18 rafts compared to that of the WT. This presumably is due to an expansion of the E2-negative midlayers of the epithelium caused by increased E6 and E7 expression (Fig. 9D). Together, these data confirm that steady-state E2 levels in the raft cultures were not affected by the mutations introduced into the HPV18 genome. Collectively, these data demonstrate that CTCF recruitment to the conserved site within the E2 ORF is important in the regulation of viral oncoprotein expression in the differentiation-dependent life cycle through a mechanism that does not involve aberrant E2 protein expression.

### CTCF controls RNA splicing of early viral transcripts.

**FIG 10:**
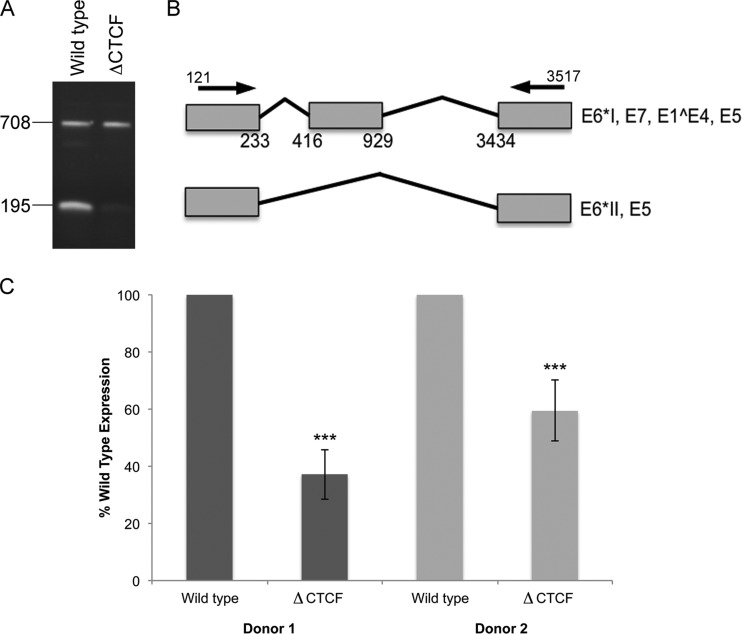
Loss of CTCF binding causes aberrant splicing of early transcripts. RNA extracted from 14-day-old raft cultures was converted to cDNA and amplified between nucleotides 121 and 3517. (A) The products were gel purified and sequenced. (B) Graphical representation of the identified products. (C) The 195-bp product was quantified using ImageJ, and relative amounts were normalized to wild-type levels for each donor. The data shown represent the means and standard errors of RNA extracted from 3 independent raft cultures from each donor (donor 1, *P* = 0.0008 [***]; donor 2, *P* = 0.0095 [***]).

A diverse range of early transcripts is expressed from the HPV genome as a result of numerous alternative splicing events (14, 15). Alterations in the splicing events that are important in early gene expression in HPV infections could have a dramatic effect on the expression of early proteins and their truncated forms (E6*I, E6*II, and E6*III) (14). Given its previously described role in the control of RNA splicing (21), CTCF binding to the E2 ORF could affect splicing of the early transcripts and viral oncoprotein expression. To test this hypothesis, RNA was extracted from raft cultures harvested at day 14 and from early transcripts amplified by RT-PCR with primer pairs that were designed previously to identify the specific splicing events that occur within the early region of the HPV18 genome (14, 15). Amplification with a 5′ primer that anneals at nucleotide 121, upstream of the first splice donor site at nucleotide 233, and 3′ primer that anneals at nucleotide 3517, downstream of the five splice acceptor sites in the early region of HPV18 at nucleotides 416, 2779, 3434, 3465, and 3506 (14, 15), was used to detect any major splicing events that occur in the early region of the HPV18 genome. Amplification of RNA from WT HPV18 rafts resulted in two major products, with some minor products visible (Fig. 10A). As previously described (14), the two major products of 708 and 195 bp were identified by sequencing and shown to be spliced at 233^416 and 929^3434 and at 233^3434, respectively (Fig. 10B). Both of these products were consistently expressed in five raft cultures from each individual donor line of WT HPV18 HFKs. Of note, the 195-bp product, spliced between nucleotides 233 and 3434, was significantly reduced in and, in some cases, absent from the ΔCTCF HPV18 raft cultures (Fig. 10A and C). This is in contrast to the increase in unspliced transcript in the ΔCTCF HPV18 rafts (Fig. 9A and B). Therefore, a significant reduction in production of the short mRNA species (233^3434 spliced product) could result in the observed increase in unspliced E6E7 transcripts. Further analysis of viral transcripts revealed that splicing events at nucleotides 233^416 and 929^3434 were not altered by the loss of CTCF binding (Table 3). These experiments demonstrate that the loss of CTCF binding at position 2989 within the HPV18 genome results in a significant alteration in splice site usage, with the specific loss of 233^3434 spliced products in the early transcripts expressed.

**TABLE 3:** Analysis of splicing events in early transcripts produced in organotypic raft culture^*a*^

Primers	Splice(s)	Product size (bp)	Fold change				Inferred ORFs
			Donor 1	*P*	Donor 2	*P*	
121/295	Unspliced Early	175	1.65 ± 0.27^*b*^	0.03	2.47 ± 0.57	0.03	E6, E7
E1F1/E4R	929^3434	190	1.049 ± 0.086	0.58	1.058 ± 0.26	0.83	E1^E4, E5
121/3517	233^416, 929^3434	708	1.03 ± 0.1	0.75	0.98 ± 0.008	0.07	E6*I, E7, E1^E4, E5
121/3517	233^3434	195	0.37 ± 0.087^*c*^	0.0008	0.59 ± 0.11	0.009	E6*II, E5

a RNA extracted from 14-day-old raft cultures was converted to cDNA and amplified with the indicated primer pairs. The fold change in transcript level compared to that of wild-type HPV18 rafts is shown as the means and standard errors from three independent repeats. Significance (*P*) was calculated using Student's *t* test.

b Significant increase in expression levels.

c Significant decrease in expression levels compared to wild type.

## DISCUSSION

This study aimed to identify CTCF binding sites within the genomes of various HPV types and to understand the function of CTCF in the virus life cycle. *In silico* predictions were used to identify potential CTCF binding sites, a high frequency of which bound CTCF *in vitro*. The relative position of many of the CTCF binding sites is conserved. A cluster of CTCF binding sites was identified in the late gene region of all of the HPV types tested, and binding within the E2 ORF appears to be conserved in the high-risk types, indicating that recruitment of CTCF to this region is related to the ability of the virus to induce cellular transformation. This suggests that the recruitment of CTCF to these regions was an early evolutionary event and that CTCF is important for the virus life cycle. Furthermore, the frequency of CTCF binding sites within the genomes of the HPV types analyzed in this study show an enrichment of sites compared to the frequency of binding sites within the human genome (20).

In contrast to the binding of CTCF within the E2 ORF in HPV16 and HPV18, CTCF recruitment within the late gene region was not detected in genome-containing cells. The conservation of the CTCF binding site cluster in the late gene region suggests that recruitment of CTCF to the late region is important for a defined point in the HPV life cycle. During submission of the manuscript, we became aware of a study by Metha et al. in which CTCF was shown to associate with the sites within the L2 gene of the late gene region of HPV31. Loss of CTCF binding to the HPV31 L2 gene appears to prevent viral genome amplification (K. Metha, V. Gunasekharan, A. Satsuka, and L. Laimins, submitted for publication). However, our data show that CTCF does not bind within the late gene region in HPV16 and HPV18 in cells grown in monolayer culture. It is possible that CTCF recruitment to this region is promoted by cellular differentiation, and this is important for capsid protein expression or viral genome amplification. Differentiation-induced loss of CpG methylation in the late region of episomal HPV16 genomes has been reported (46). CpG methylation can negatively regulate CTCF binding (47), making this method of regulation of CTCF recruitment to the late gene region of the HPV genome in differentiating epithelium a plausible hypothesis.

Several host cell proteins are recruited to the HPV genome to regulate transcriptional control. The binding of host cell transcription factors to sequences within the URR to control early gene transcription has been well characterized. Transcriptional regulators, such as AP1 (48), SP1 (49), TFIID (50), TBP (51), NF1, and Oct-1 (52), have defined binding sites within the URR of all HPV types analyzed. Many other transcriptional regulators are recruited by association with the E2 protein, including Brd4 (53), TaxBP1 (54), p300, and CBP (55). In contrast, very few host or viral proteins have been shown to bind specifically to the HPV genome outside the URR, although evidence of C/EBPβ, Oct-1, and YY1 binding to sequences upstream of the late promoter within the E7 ORF in HPV18 has been reported (56–58). The recruitment of CTCF to a binding site that exists within the E2 ORF is, to our knowledge, the first description of a cellular factor recruited to a specific binding site outside the URR or late promoter regions to control viral gene expression.

Mutation of the CTCF binding site within the E2 ORF of HPV18 has highlighted an important function of CTCF in the HPV life cycle. Growth of cells in organotypic raft culture was affected by abrogation of CTCF binding, and we noted a significant increase in cellular proliferation coupled with enhanced E6 and E7 protein expression. These data provide evidence that loss of CTCF binding within the E2 ORF enhances E6 and E7 expression in differentiating cells, prolonging the proliferative potential of cells in the middle and upper layers of the stratified epithelium. It is interesting that although we observed an increase in cell cycle entry in the ΔCTCF HPV18 raft cultures, we did not observe an increase in mitotic entry. One possible explanation for this is that the raft cultures were harvested at 14 days when the epithelia were fully differentiated. It is possible that an increase in mitosis occurs as the epithelium is developing and that in a fully differentiated epithelium, the cells are more likely to arrest in G_2_ than progress through mitosis. Importantly, there were no discernible effects on the overall expression of E2, although expansion of the midlayers of the epithelium resulted in an apparent delay in E2 expression. In addition, viral genome replication and amplification were unaffected, suggesting that E1 protein levels were unaffected. This suggests that HPV18 and perhaps other oncogenic HPV types have evolved to bind CTCF in this region to regulate balanced and controlled E6 and E7 expression in the context of a productive infection. Interestingly, CTCF does not appear to bind to the site within the E2 ORF in integrated sequences in HeLa cells (59), even though three copies of the binding site exist (60). It is possible that CpG methylation prevents CTCF binding to this site in HeLa cells, as previously reported (47, 61), and it is tempting to speculate that the apparent loss of CTCF binding in integrated HPV18 genomes in HeLa cells contributes to the high E6 and E7 expression in these cells.

It should be noted that CTCF binding sites have been identified within the genomes of large DNA viruses, such as Epstein-Barr virus (EBV) and Kaposi's sarcoma-associated herpesvirus (KSHV). Mutation of sites to prevent CTCF binding has demonstrated that CTCF determines latency in these viruses by blocking epigenetic silencing of latency-associated promoter elements and mediating long-range interactions within the viral genome (62–68). This, in part, is thought to be through CTCF-dependent regulation of nucleosome organization and control of RNA polymerase II recruitment to the latency control region (69, 70). Whether CTCF binding within the E2 ORF of the HPV genome directly controls E6E7 transcript production through similar mechanisms currently is being explored.

CTCF binding within the host genome controls cotranscriptional alternative splicing events by creating a roadblock to processing RNA polymerase II and promoting inclusion of weak upstream exons (21). Therefore, we analyzed splicing events that occur in the HPV early transcripts in differentiating epithelium and demonstrated a significant increase in the unspliced early transcript that encodes the E6 and E7 oncoproteins. In addition, the transcript spliced directly at 233^3434 was markedly reduced in ΔCTCF HPV18 rafts, while the abundance of all other spliced products was unchanged. The transcript spliced at 233^3434 could be used as a template for translation of E6*II and E5 (14). Multiple transcripts that are abundantly and equally expressed in our WT and ΔCTCF rafts potentially encode E5 protein, making it unlikely that E5 expression is affected by loss of CTCF binding. Whether the loss of E6*II expression contributes to the phenotype observed in our mutant HPV18 HFK rafts remains to be determined.

Our data suggest that CTCF recruitment to the E2 ORF binding site is a control mechanism for the expression of unspliced and alternatively spliced early transcripts in the HPV life cycle. It is interesting that the current model of CTCF-mediated splicing regulation predicts that DNA-bound CTCF pauses RNA polymerase II progression and promotes the inclusion of weak upstream exons by allowing the splicing machinery more time to process the nascent RNA strand (21). Our data support a role for CTCF in directing splicing events but suggests that the function of CTCF in this process is more complex than the current model predicts. In our physiologically relevant model system, loss of CTCF binding results in both increased levels of unspliced transcripts and a complex alteration of splice site usage upstream of the CTCF binding site. Further study of CTCF in the regulation of RNA processing likely will highlight novel functions of CTCF in gene expression regulation.
